# Production of a Novel Fucoidanase for the Green Synthesis of Gold Nanoparticles by *Streptomyces* sp. and Its Cytotoxic Effect on HeLa Cells

**DOI:** 10.3390/md13116818

**Published:** 2015-11-12

**Authors:** Panchanathan Manivasagan, Junghwan Oh

**Affiliations:** 1 Marine-Integrated Bionics Research Center, Pukyong National University, Busan 608-737, Korea; E-Mail: manimaribtech@gmail.com; 2 Department of Biomedical Engineering and Center for Marine-Integrated Biotechnology (BK21 Plus), Pukyong National University, Busan 608-737, Korea

**Keywords:** fucoidan, fucoidanase, *Streptomyces*, gold nanoparticles, HeLa cells

## Abstract

Marine actinobacteria-produced fucoidanases have received considerable attention as one of the major research topics in recent years, particularly for the medical exploitation of fucoidans and their degradation products. The present study describes the optimization and production of a novel fucoidanase for the green synthesis of gold nanoparticles and its biological applications. The production of fucoidanase was optimized using *Streptomyces* sp. The medium components were selected in accordance with the Plackett-Burman design and were further optimized via response surface methodology. The fucoidanase was statistically optimized with the most significant factors, namely wheat bran 3.3441 g/L, kelp powder 0.7041 g/L, and NaCl 0.8807 g/L, respectively. The biosynthesized gold nanoparticles were determined by UV-vis spectroscopy and were further characterized by X-ray diffraction analysis, Fourier transform infrared spectroscopy, field emission scanning electron microscopy, energy dispersive X-ray analysis, and high-resolution transmission electron microscopy. Furthermore, the biosynthesized gold nanoparticles exhibited a dose-dependent cytotoxicity against HeLa cells and the inhibitory concentration (IC_50_) was found to be 350 µg/mL at 24 h and 250 µg/mL at 48 h. Therefore, the production of novel fucoidanase for the green synthesis of gold nanoparticles has comparatively rapid, less expensive and wide application to anticancer therapy in modern medicine.

## 1. Introduction

Marine actinobacteria-produced fucoidanases have attracted a great deal of attention in recent years because there are many advantages in the medical exploitation of the fucoidans and their degradation products [1]. Marine algae have been recognized as a rich source of biological macromolecules that are of potential interest to various industrial applications, such as food, cosmetics, and pharmacology [2]. Fucoidan is a sulfated polysaccharide extracted from a marine brown algae; it has a high molecular weight and is highly sulfated. Fucoidans have diverse biological activities such as anticoagulant [3], antitumor [4], antibacterial [5], antiviral [6], anti-inflammatory [7], and antithrombotic [8] activities. In recent years, the interest in fucoidan from different sources, its structure, and its possible biological applications has increased. In the last decade, the research on fucoidanases has come into focus and is gradually developing.

As the fucoidan molecule is too large to be used as a clinical drug, investigators are interested in synthesizing a low molecular weight fucoidan (LMWF) that could obviously decrease the side effects and antigenicity caused by fucoidan [9]. Fucoidanase (E.C. 3.2.1.44) can hydrolyze fucoidan to produce sulfated LMWF without the removal of its side substitute groups because of its substrate hydrolysis property. There are only a very few studies on isolation and characterization of fucoidanases [10]. To date, fucoidanases are reported only from marine organisms such as bacteria [11,12,13], fungi [9,14,15], and invertebrates [10]. However, the fucoidanases produced by these organisms have low activities [16]. Therefore, there is an interest in establishing a process to produce this enzyme at elevated levels.

Gold nanoparticles have gained attention as one of the major emerging areas of research in recent years because of their unique and intense plasmon resonance in the visible range and their application in biomedical sciences [17]. The marine actinobacterial synthesis of gold nanoparticles has good potential to develop simple, cost-effective, and eco-friendly methods for the production of important bionanomaterials [18,19]. Maine actinobacteria, particularly *Streptomyces* sp., are well known for their unique ability to produce a wide variety of novel secondary metabolites, such as antibiotics, immunosuppressors, and many other biologically active compounds [20,21,22]. *Streptomyces* sp. are widely used in the pharmaceutical and enzyme industries and are well known for industrial manufacturing purposes. The present study involves the optimization and production of a novel fucoidanase for the biosynthesis of gold nanoparticles by *Streptomyces* sp., and their biomedical applications. To the best of our knowledge, this marine actinobacterium *Streptomyces* sp. has never been used previously for the production of a novel fucoidanase for the green synthesis of gold nanoparticles and its cytotoxic effect on HeLa cells.

## 2. Results and Discussion

### 2.1. Isolation and Identification of Marine Actinobacterium

**Figure 1:**
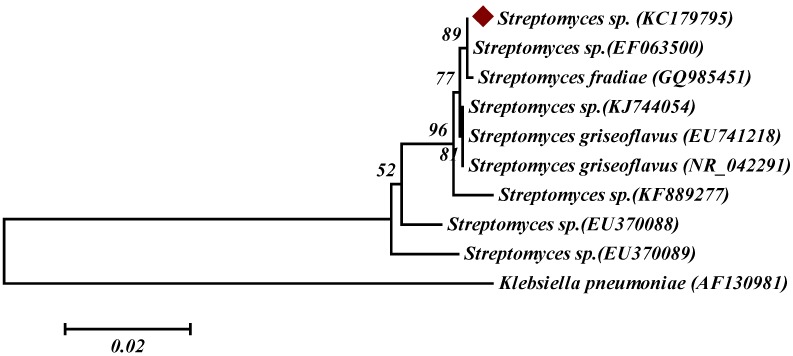
Phylogenetic tree of 10 strains of scab-causing and related *Streptomyces* sp. based on the 16S rDNA gene sequence. The numbers at the branching points are the percentages of occurrence in 1000 bootstrapped trees. The bar indicates a distance of 0.02 substitutions per site.

The marine actinobacterium *Streptomyces* sp. was isolated from the marine sediment samples collected from the Busan coast, South Korea, and was used for the production of fucoidanase. On the basis of the growth rate and high fucoidanase activity, *Streptomyces* sp. was considered as an effective producer of fucoidanase. The active producer was identified using cultural, morphological, biochemical, physiological characteristics, and 16S rDNA sequence (Figure 1). The isolate was identified as *Streptomyces* sp., and the 16S rDNA sequence of actinobacterium *Streptomyces* was deposited in NCBI (Accession No. KC179795).

### 2.2. Fucoidanase Production

**Figure 2:**
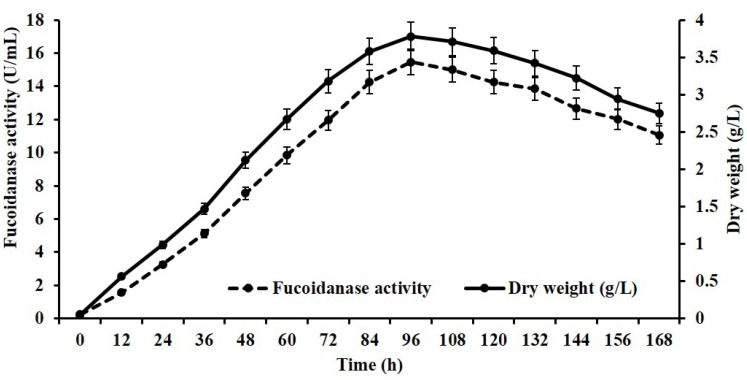
Growth and fucoidanase activity of *Streptomyces* sp.

The correlation between fucoidanase production and culturing time may differ among different organisms [9]. It was observed that an extension in cultivation period resulted in an increase in cell growth with a concomitant increase in fucoidanase activity (Figure 2). The production of the fucoidanase was associated with cell growth, increased during the logarithmic growth, and reached its maximum fucoidanase activity in the stationary phase (96 h). Similar to our findings, fucoidanase production by both *Dendryphiella arenaria* TM94 [9] and *Pseudoalteromonas citrea* [11] was synchronous with cell growth and reached the maximum concentration in the stationary phase of the cell.

### 2.3. Plackett-Burman (PB) Design

The Plackett-Burman (PB) design is a powerful technique for screening and evaluating the important variables that influence the response. The experiment was conducted in 12 runs to study the effect of the selected variables. Statistical analysis of the responses is represented in Table 1; Table 2 shows the results of the screening experiments using PB design. The adequacy of the model was calculated, and the variables evidencing statistically significant effects were screened via Student’s *t*-test for analysis of variance (ANOVA) (Table 1). Factors evidencing *p*-values of less than 0.05 were considered to have significant effects on the response and were, therefore, selected for further optimization studies. Regression analysis was performed on the results and a first-order polynomial Equation (1) was derived, representing fucoidanase production as a function of the independent variables.

*Y* = 0.5909 + 0.5008*X*_1_ − 1.5370*X*_2_ − 5.0583*X*_3_ + 108*X*_4_ + 71.5*X*_5_ + 5.6778*X*_6_ (1)

The magnitude of the effects indicated the level of the significance of the variables on fucoidanase production. Among the variables studied *X*_1_, *X*_2_, and *X*_6_ were identified as the most significant variables influencing the fucoidanase production. Thus, the three variables, wheat bran (*X*_1_), kelp powder (*X*_2_), and NaCl (*X*_6_) were selected and their optimal levels were identified using RSM.

**Table 1:** The concentrations of involved variables at different levels and the regression analysis of the Plackett*-*Burman design.

No.	Variables	Concentration (g/L)		Effect	Coefficients	Standard Error	*t*-Value	*p*-Value
		−1 Level	+1 Level					
*X*_1_	Wheat bran	1.0	5.0	2.003	1.002	0.1868	5.36	0.003 ^a,^**
*X*_2_	Kelp powder	0.1	1.0	−1.383	−0.692	0.1868	−3.70	0.014 ^b,^*
*X*_3_	Glucose	0.1	0.5	−2.023	−1.012	0.1868	−5.42	0.003 ^b,^**
*X*_4_	NaNO_3_	0.01	0.05	4.320	2.160	0.1868	11.56	0.000 ^a,^**
*X*_5_	MgSO_4_·7H_2_O	0.01	0.05	2.860	1.430	0.1868	7.66	0.001 ^a,^**
*X*_6_	NaCl	0.1	1.0	5.110	2.555	0.1868	13.68	0.000 ^a,^**

*R*^2^ = 98.90%; *R*^2^_Adj_ = 97.59%; *R*^2^_Pre_ = 93.69%; Non-significant at *p* < 0.05; ^a^ Significant positive effect; ^b^ Significant negative effect; * <0.05; ** <0.01.

**Table 2:** Plackett*-*Burman design for the screening of critical media components involved in fucoidanase activity.

Runs	Real Levels (Coded Levels)/Concentrations (g/L)						Fucoidanase Activity (U/mL)	
	*X*_1_	*X*_2_	*X*_3_	*X*_4_	*X*_5_	*X*_6_	Observed ^a^	Predicted
1	5 (+1)	0.1 (−1)	0.5 (+1)	0.01 (−1)	0.01 (−1)	0.1 (−1)	2.83	2.78
2	5 (+1)	1.0 (+1)	0.1 (−1)	0.05 (+1)	0.01 (−1)	0.1 (−1)	7.71	7.74
3	1 (−1)	1.0 (+1)	0.5 (+1)	0.01 (−1)	0.05 (+1)	0.1 (−1)	2.42	2.25
4	5 (+1)	0.1 (−1)	0.5 (+1)	0.05 (+1)	0.01 (−1)	1.0 (+1)	12.56	12.21
5	5 (+1)	1.0 (+1)	0.1 (−1)	0.05 (+1)	0.05 (+1)	0.1 (−1)	10.62	10.60
6	5 (+1)	1.0 (+1)	0.5 (+1)	0.01 (−1)	0.05 (+1)	1.0 (+1)	8.91	9.36
7	1 (−1)	1.0 (+1)	0.5 (+1)	0.05 (+1)	0.01 (−1)	1.0 (+1)	8.25	8.82
8	1 (−1)	0.1 (−1)	0.5 (+1)	0.05 (+1)	0.05 (+1)	0.1 (−1)	8.39	7.95
9	1 (−1)	0.1 (−1)	0.1 (−1)	0.05 (+1)	0.05 (+1)	1.0 (+1)	14.86	15.09
10	5 (+1)	0.1 (−1)	0.1 (−1)	0.01 (−1)	0.05 (+1)	1.0 (+1)	12.81	12.77
11	1 (−1)	1.0 (+1)	0.1 (−1)	0.01 (−1)	0.01 (−1)	1.0 (+1)	7.37	6.52
12	1 (−1)	0.1 (−1)	0.1 (−1)	0.01 (−1)	0.01 (−1)	0.1 (−1)	2.13	2.80

^a^ The observed values of fucoidanase production were the mean values of duplicates.

### 2.4. Response Surface Methodology (RSM)

The significant independent variables (wheat bran (*X*_1_), kelp powder (*X*_2_), and NaCl (*X*_6_)) were used to determine the optimum levels of these parameters based on the above results. The Box-Behnken design with three factors and three levels, including five replicates at the center point, was used for fitting a second-order response surface (Table 3). The design matrix and the corresponding experimental data are shown in Table 3. The results were analyzed by standard ANOVA, and following quadratic regression equations were obtained in terms of fucoidanase production. Using the designed experimental data, the polynomial Model (2) for fucoidanase yield *Y* was regressed by only considering the significant terms, and is shown as follows:

*Y =* −2.7321 *+* 5.3828*X*_1_*+* 27.3222*X*_2_ − 5.5679*X*_6_*+* 0.2333*X*_1_*X*_2_ − 0.6361*X*_1_*X*_6_*+* 4.2222*X*_2_*X*_6_*−* 0.7547*X*^2^_1_ − 22.9444*X*^2^_2_*+* 8.2284*X*^2^_6_ (2)

where *Y* is the predicted fucoidanase yield, *X*_1_ is wheat bran, *X*_2_ is kelp powder and *X*_6_ is NaCl.

The statistical significance of the equation was evaluated by performing *F*-test and ANOVA with the Design Expert 7.0 software. As shown in Table 4, the model *F*-value was 1439.22, and the *F*-value for lack of fit was 1.10 (Table 4). The high *F*-value and non-significant lack of fit indicate that the model is a good fit. The *p*-values of the model (<0.0001) and the lack of fit (0.4456) also suggested that the obtained experimental data was a good fit with the model. The fit of the model was also checked by the determination coefficient (*R*^2^). Table 4 shows the ANOVA (*F*-test) for this experiment. The value of *R*^2^ was 0.9995 for fucoidanase yield, which explained 99.95% of the response variability. This value indicated that the accuracy and general ability of the polynomial model was good. The adjusted determination coefficient (*R*^2^_Adj_ = 0.9988) and predicted determination coefficient (*R*^2^*_Pre_* = 0.9956) were also satisfactory to confirm the significance of the model. Analysis of the response trends using the model was considered to be reasonable.

**Table 3:** Experimental design and results of the Box*-*Behnken optimization experiment.

Trials	*X*_1_	*X*_2_	*X*_6_	Fucoidanase Activity (U/mL)	
				Observed ^a^	Predicted
1	1	0.10	0.55	3.71	3.73
2	5	0.10	0.55	5.83	5.84
3	1	1.00	0.55	7.92	7.91
4	5	1.00	0.55	10.88	10.86
5	1	0.55	0.10	9.89	9.81
6	5	0.55	0.10	13.56	13.48
7	1	0.55	1.00	14.38	14.46
8	5	0.55	1.00	15.76	15.85
9	3	0.10	0.10	8.51	8.57
10	3	1.00	0.10	11.36	11.46
11	3	0.10	1.00	10.47	10.37
12	3	1.00	1.00	16.74	16.68
13	3	0.55	0.55	14.52	14.75
14	3	0.55	0.55	14.83	14.75
15	3	0.55	0.55	14.79	14.75
16	3	0.55	0.55	14.81	14.75
17	3	0.55	0.55	14.80	14.75

^a^ The observed values of fucoidanase production were the mean values of duplicates.

**Table 4:** Analysis of variance (ANOVA) for predictive equation for fucoidanase production by *Streptomyces* sp.

Source	Sum of Squares	*DF*	Mean Square	*F*-Value	*p*-Value > *F*
Model	226.45	9	25.16	1439.22	<0.0001 **
*X*_1_	12.83	1	12.83	733.73	<0.0001 **
*X*_2_	42.23	1	42.23	2415.50	<0.0001 **
*X*_6_	24.61	1	24.61	1407.44	<0.0001 **
*X*_1_*X*_2_	0.18	1	0.18	10.09	<0.0156 *
*X*_1_*X*_6_	1.31	1	1.31	74.99	<0.0001 **
*X*_2_*X*_6_	2.92	1	2.92	167.26	<0.0001 **
*X*^2^_1_	38.37	1	38.37	2194.81	<0.0001 **
*X*^2^_2_	90.90	1	90.90	5199.32	<0.0001 **
*X*^2^_6_	11.69	1	11.69	668.69	<0.0001 **
Residual	0.12	7	0.017		
Lack-of-fit	0.055	3	0.018	1.10	0.4456 ^NS^
Pure error	0.067	4	0.017		
Cor total	226.57	16			

*R*^2^*=* 0.9995; CV = 1.11; *R*^2^_Adj_ *=* 0.9988; *R*^2^_Pre_ *=* 0.9956; DF—degree of freedom; *F*—Fischer’s test; *P*—Probability value; * <0.05; ** <0.01; NS = non-significant.

The model coefficients calculated by regression analysis for each variable are shown in Table 4, where the regression coefficients of all linear, quadratic terms, and two cross-products are significant at a 1% level.

**Figure 3:**
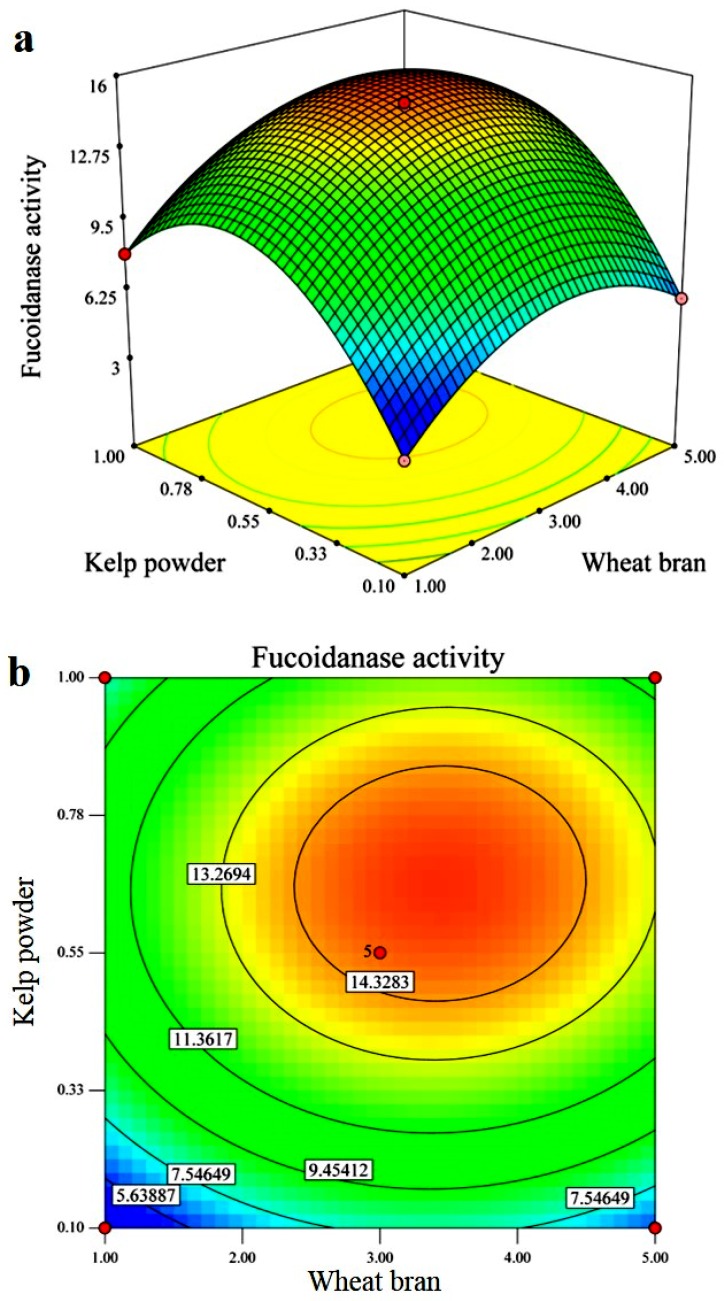
Response surface plots (**a**) and contour plots (**b**) for the fucoidanase activity optimization. The interaction between kelp powder and wheat bran.

Three dimensional response surface plots (Figure 3a) and 2D contour plots (Figure 3b) of fucoidanase production graphically representing regression equations were used to demonstrate relationships between the response and experimental levels of each variable. Each figure presents the effect of two factors, whereas the other factor was held at zero level. As shown in the surface plots, there was an interaction between each pair of variables. All the interaction between the selected three variables was significant.

According to the canonical analysis, the results predicted by the model showed that the maximum fucoidanase production could be achieved when the wheat bran (*X*_1_), kelp powder (*X*_2_) and NaCl (*X*_6_) were set at 3.3441, 0.7041, and 0.8807 g/L, respectively. The maximum predicted value of fucoidanase yield obtained was 17.4640 U/mL.

To confirm the optimization results, using the suggested medium components and conditions, experiments were performed in triplicate. Under these suggested conditions, the mean value of the fucoidanase yield was 16.74 U/mL, which was in agreement with the predicted value. This optimization strategy led to the enhancement of fucoidanase from 3.71 U/mL to 16.74 U/mL. The models developed were considered to be accurate and reliable for predicting the production of fucoidanase by *Streptomyces* sp.

### 2.5. Biosynthesis and Characterization of Gold Nanoparticles

**Figure 4:**
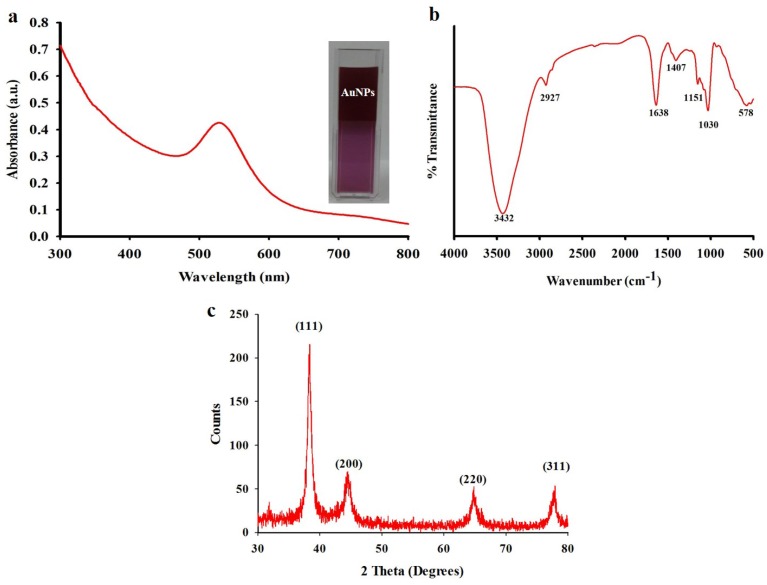
(**a**) UV-vis spectral analysis of biosynthesized gold nanoparticles; (**b**) Fourier transform infrared spectroscopy (FTIR) spectra of biosynthesized gold nanoparticles; (**c**) XRD pattern of biosynthesized gold nanoparticles.

The purified fucoidanase was added to an aqueous solution of gold chloride at 80 °C for 30 min, resulting in a color changing of solution that indicates the formation of gold nanoparticles observed by UV-vis spectroscopy. The biosynthesis of gold nanoparticles was evidenced through the appearance of an intense pinkish ruby red color because of the reduction of Au^+^ and formation of stable gold nanoparticles. The results indicated that the reaction solution showed an absorption peak at 531 nm attributed to the surface plasmon resonance band of the gold nanoparticles (Figure 4a). Various research groups have reported a change in the color from yellowish to pinkish ruby red after biotransformation during the biosynthetic process [19,23,24]. Although the common underlying mechanism involved in biosynthesis is the reduction of gold ions (Au^+3^) to form gold nanoparticles, it has been postulated that the enzymes secreted by microorganisms play an important role in the bioreduction of metal ions, leading to nanoparticle nucleation and growth [23]. Despite the enormous number of reports on microbially mediated gold nanoparticles synthesis, the mechanistic characteristics have not been established and need to be reported in depth [18,19,23].

Fourier transform infrared spectroscopy (FTIR) was used to characterize the functional groups, which may play an important role in stabilizing the gold nanoparticles. FTIR spectra of the biosynthesized gold nanoparticles, as shown in Figure 4b, can offer information regarding the chemical change of the functional groups involved in the reduction. The FTIR spectrum of the biosynthesized gold nanoparticles showed characteristic bands at 3432, 2927, 1638, 1407, 1151, 1030, and 578 cm^−1^. The strong broad absorbance at 3432 cm^−1^ corresponds to O–H stretching vibrations of the hydroxyl functional group in alcohols and phenolic compounds. The intense medium absorbance at 2927 cm^−1^ corresponds to C–H stretching of alkanes, and 1638 cm^−1^ is characteristic of N–H bending of 1 º amines. The bands at 1407 cm^−1^ correspond to C–C stretching of aromatics. The band at 1151 and 1030 cm^−1^ can be assigned to the C–N stretching of aliphatic amines group. The medium bands at 578 cm^−^^1^ correspond to the C–Br stretching of the alkyl halides. A previous report reveals that the hydroxyl group (O–H) has a strong ability to interact with gold nanoparticles [18,25,26].

The crystalline nature of biosynthesized gold nanoparticles was further confirmed from XRD analysis. The XRD patterns of synthesized gold nanoparticles are shown in Figure 4c. The four intense diffraction peaks were observed at 2Ө values of 38.13°, 44.43°, 64.66°, and 77.66°, corresponding to the (111), (200), (220), and (311) reflection of the crystalline metallic gold, respectively (JCPDS No. 04-0784). Earlier studies have reported the crystalline nature of biosynthesized gold nanoparticles using marine organisms [27,28].

**Figure 5:**
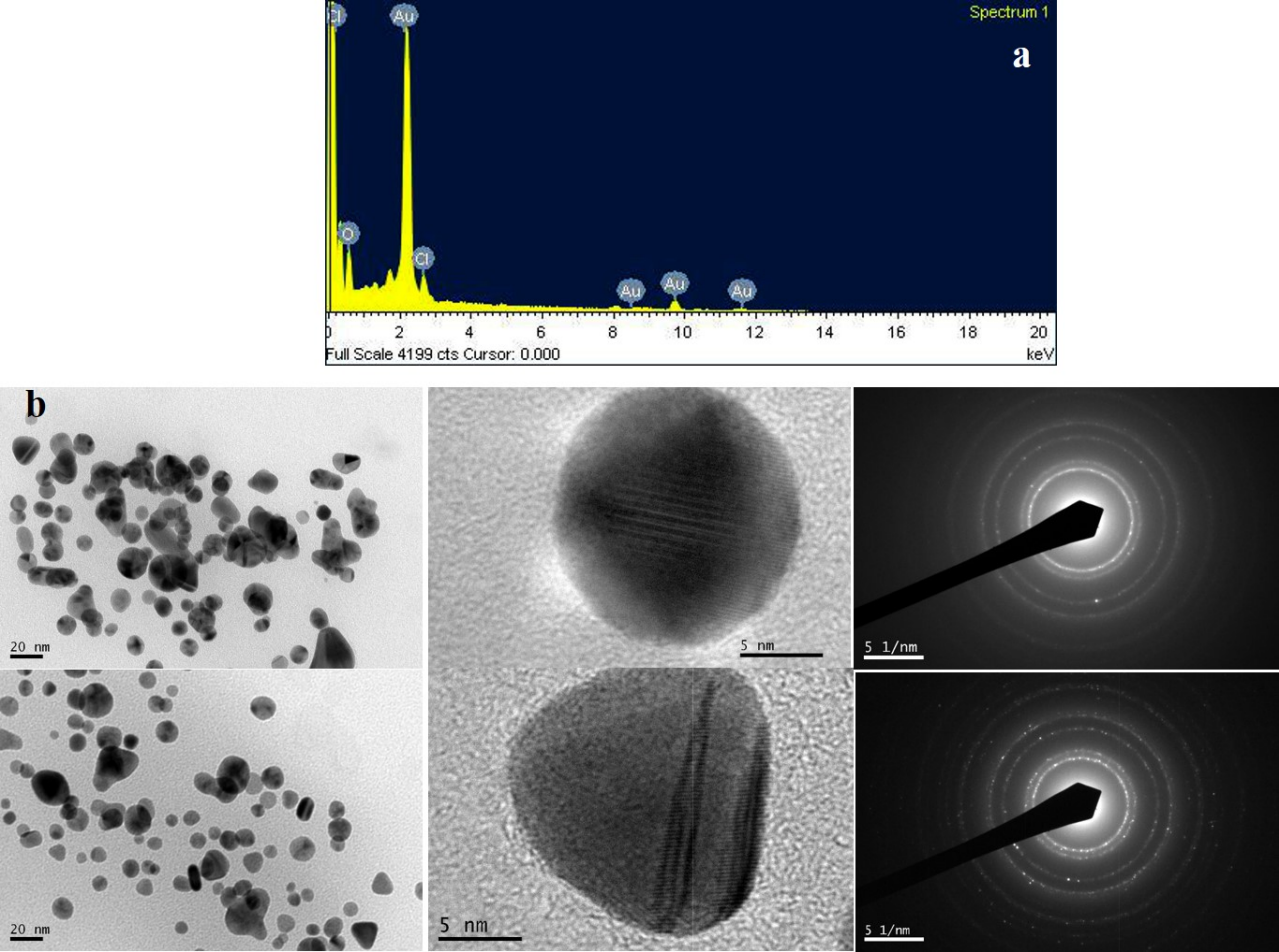
(**a**) Energy dispersive X-ray spectrum of biosynthesized gold nanoparticles; (**b**) High resolution transmission electron microscopy (HRTEM) images of biosynthesized gold nanoparticles (20 nm and 5 nm scale) and selected area electron diffraction (SAED) pattern.

Field emission scanning electron microscopy (FESEM) clearly shows the presence of the biosynthesized gold nanoparticles, which were confirmed to be gold by energy dispersive X-ray analysis (EDXA). EDXA (Figure 5a) spectrum represents the signal from Au^+^ ions together with Cl and O. Signals appear from Cl and O because of the X-ray emission from the biological macromolecules like proteins/enzymes bound to the nanoparticles or in the vicinity of the particles. The size and morphology of the biosynthesized gold nanoparticles was confirmed by high-resolution transmission electron microscopy (HRTEM) analysis. Figure 5b shows the mixture of gold nanospheres and gold nanoprisms (mostly truncated triangles). Most of the nanoparticles formed by this green method are well separated, and have a diameter in the range of 10–50 nm with an average particle size of 39 ± 2 nm. According to the face-centered cubic structure of biosynthesized gold nanoparticles, the spots in the selected area electron diffraction (SAED) pattern are indexed and reveal that the particles are single, and crystalline in nature. Previous studies report that HRTEM images for the biosynthesized gold nanoparticles are spherical in shape with an average size ranging from 10 to 100 nm [29,30].

### 2.6. In Vitro Anticancer Activity of Gold Nanoparticles

#### 2.6.1. Cell Viability

**Figure 6:**
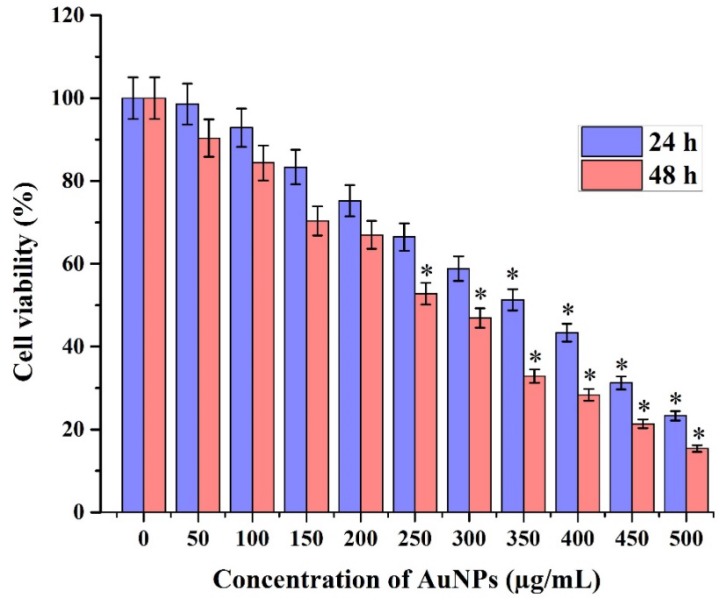
MTT assay results confirming the *in vitro* cytotoxicity effect of gold nanoparticles against the HeLa cells for 24 h and 48 h respectively. Data is expressed as mean ± SD of three experiments. Percentage of cytotoxicity is expressed relative to untreated controls (* significant *p* < 0.05).

Gold nanoparticles as novel agents for cancer therapy are gaining greater demand in medical applications. At the same time, there are only limited studies on the cytotoxic activity of biosynthesized gold nanoparticles against cancer cell lines. MTT assay was used to assess the effect of gold nanoparticles on the proliferation of HeLa cells. In the present study, we investigated that the induction of apoptosis could be the possible mechanism for the antiproliferative activity of biosynthesized gold nanoparticles. The dose-dependent cytotoxicity was observed in gold nanoparticle-treated HeLa cells. Fifty percent of cell death, determining the inhibitory concentration (IC_50_) value of biosynthesized gold nanoparticles against HeLa cells holds at 350 µg/mL in 24 h and 250 µg/mL in 48 h (Figure 6a). The experimental results clearly proved the excellent anticancer activity of gold nanoparticles against the HeLa cell line [31,32].

#### 2.6.2. Morphological Observation

**Figure 7:**
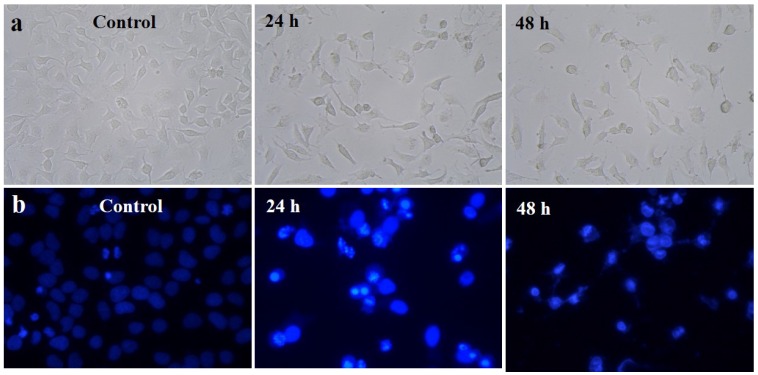
(**a**) Phase contrast microscopic images of gold nanoparticles induced gross cytomorphological changes and growth inhibition at different time (24 h and 48 h) intervals on the HeLa cells; (**b**) 4′,6-diamidino-2-phenylindole dihydrochloride (DAPI) staining shows apoptotic and necrotic cell death due to the cytotoxicity of biosynthesized gold nanoparticles (24 h and 48 h).

The diverse morphological alteration was observed in gold nanoparticles-treated HeLa cells; however, no such effects were seen in untreated cells. It was shown that the morphological variations were observed such as the loss of membrane integrity, inhibition of cell growth, cytoplasmic condensation, and cell clumping, indicating that the gold nanoparticles treated HeLa cells undergone cell death, whereas the non-treated cell were active (Figure 7a).

#### 2.6.3. 4′,6-Diamidino-2-Phenylindole Dihydrochloride (DAPI) for Nuclear Staining

**Scheme 1:**
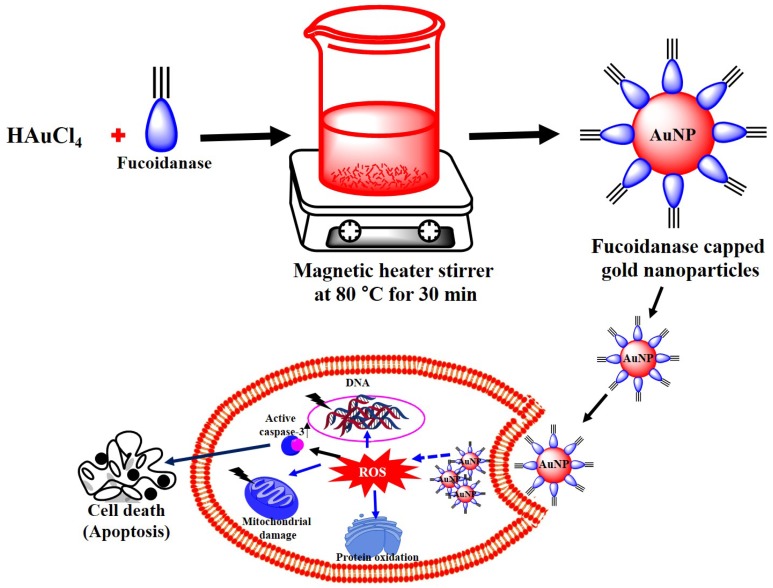
Overall scheme for the production of novel fucoidanase for the green synthesis of gold nanoparticles and their *in vitro* anticancer activity.

DAPI is a popular nuclear counterstain; thus, the gold nanoparticle-induced nuclear fragmentation was observed by DAPI staining. The untreated cells showed normal nuclei (smooth nuclear), whereas after treatment of HeLa cells with gold nanoparticles, the apoptotic nuclei (condensed and fragmented chromatin) were observed as shown in Figure 7b. Nuclear morphology analysis showed characteristic apoptotic changes, such as chromatin condensation, fragmentation of the nucleus, and formation of apoptotic bodies in the HeLa cells. The Scheme 1 represents the production of novel fucoidanase for the green synthesis of gold nanoparticles and their role as an anticancer agent through the formation of reactive oxygen species (ROS) and apoptotic pathway. Interestingly, some studies have reported that gold nanoparticles can also induce DNA damage and apoptosis in cancer cells [27,29].

## 3. Materials and Methods

### 3.1. Chemicals

Fucoidan from *Fucus vesiculosus* and gold (III) chloride trihydrate (HAuCl_4_∙3H_2_O) were obtained from Sigma-Aldrich (St. Louis, MO, USA). All media components were purchased from Lab M Limited (Bury, UK). All chemicals were of analytical grade and procured from Sigma-Aldrich (St. Louis, MO, USA).

### 3.2. Isolation and Identification of Marine Actinobacterium

The marine actinobacterium *Streptomyces* sp. was isolated from the marine sediment samples collected from the Busan coast (Lat 35°09′ N; Long 129°07′ E), South Korea and was identified on the basis of the 16S rDNA gene sequencing. The 16S rDNA gene was amplified by PCR using the universal primer pair 27 F 5′-AGAGTTTGATCMTGGCTCAG-3′ and 1518 R 5′-AAGGAGGTGWTCCARCC-3′. The amplified products were analyzed by electrophoresis in 0.7% (*w*/*v*) agarose gel and purified using a DNA extraction kit (RBC, Seongnam-si Gyeonggi-do, Korea). The 16S rDNA sequencing was performed using Macrogen, South Korea. The DNA sequence analysis was then performed by BLAST network services at the NCBI. The 16S rRNA gene sequences of *Streptomyces* sp. (~1513 bp) was aligned with reference sequences obtained from GenBank using ClustaL X 2.0.11. The GenBank accession number of *Streptomyces* sp. is KC179795. The phylogenetic tree was generated using the neighbor-joining method with MEGA 6.0 package [33].

### 3.3. Fucoidanase Production

Fucoidanase production was carried out following Wu *et al.* [9] with slight modification. Solid-state medium consisted of 5.0 g wheat bran, 1.0 g kelp powder, 0.5 g glucose, 0.05 g NaNO_3_, 0.05 g MgSO_4_·7H_2_O, 1.0 g NaCl, and 100% seawater. All the experiments were carried out in 500mL Erlenmeyer flasks containing 100 mL of solid-state medium. The sterile medium was inoculated with 5% of inoculum (3.1–4.7 × 10^4^ CFU/mL), incubated at 28 °C and cultivated under agitation at 180 rpm for 7 days. Samples were withdrawn at different time intervals and monitored for cell growth and fucoidanase activity. The culture broth was centrifuged at 10,000 rpm for 20 min to separate the cells that were washed twice with distilled water and dried at 65 °C to constant weight as a measurement of cell growth. The culture supernatant was used to study fucoidanase activity. All experiments were performed in triplicate for the mean calculation.

### 3.4. Enzyme Assay

#### 3.4.1. Fucoidanase Activity

Fucoidanase activity was measured by the dinitrosalicylic acid (DNS) technique [34] to estimate the release of reducing sugars using the following reaction: a mixture consisting of 0.9 mL substrate solution (1% (*w*/*v*) fucoidan from *F. vesiculosus* dissolved with 0.1 M citric acid-sodium citric buffer, pH 6.0) and 0.1 mL enzyme solution was incubated at 50 °C for 10 min, using inactivated enzyme solution as blank CK. The reaction was terminated by the addition of 1 mL DNS reagent. The mixture was kept in a boiling water bath for 7 min at 100 °C. The mixture was cooled to room temperature and 3 mL of distilled water was added. The absorbance was read at 540 nm in a UV-vis spectrophotometer. The amount of reducing sugar formed during the reaction was determined using a fucose standard plot. One unit (U) of fucoidanase activity is defined as the amount of enzyme that releases 1 μmol of fucose per minute under the assay conditions.

#### 3.4.2. Fucosidase Activity

Fucosidase activity was measured under the following conditions: the reaction mixture contained 0.9 mL substrate solution (1% ρ-nitrophenyl-α-l-fucoside (*w*/*v*) dissolved with 0.1 M citric acid-sodium citric buffer, pH 6.0) and 0.1 enzyme solution was incubated at 40 °C for 2 h. One unit (U) of fucosidase activity is defined as the amount of enzyme that releases 1 μmol ρ-nitrophenyl per minute under the assay conditions.

#### 3.4.3. Amylase Assay

Amylase activity was determined by measuring the release of reducing sugar from soluble starch. The reaction mixture contained 0.1 mL of enzyme solution and 0.9 mL of 100 mM phosphate buffer (pH 7.0) containing 1% (*w*/*v*) of soluble starch. The mixture was incubated at 60 °C for 15 min. The amount of reducing sugar level released in the mixture was determined by the DNS method [34]. The absorbance was measured at 540 nm. d-Glucose was used to obtain a standard curve. One unit (U) of enzyme activity is defined as the amount of enzyme releasing 1 µmol glucose per min under the assay conditions.

### 3.5. Experimental Design and Data Analysis

#### 3.5.1. Plackett-Burman (PB) Design

PB design, an efficient technique for medium component optimization, was employed for screening fermentation parameters that significantly influenced fucoidanase production. This model significantly decreases the number of experiments needed to effectively achieve experimental goals [35]. In the present study, six independent medium components were investigated using PB design to identify the components that significantly affected fucoidanase activity. The components included wheat bran, kelp powder, glucose, NaNO_3_, MgSO_4_·7H_2_O, and NaCl. Two concentrations, “high” and “low”, were evaluated for each medium component and designated as level +1 and level *−*1, respectively (Table 1).

The design was developed by the “Design Expert” software (Version 7.0, Stat-Ease Inc., Minneapolis, MN, USA). The PB experimental design was based on the following first-order Model (3):

(3) $$Y=\beta_{0}+\sum_{i}^{k}\beta_{i}X_{i}$$

where *Y* represents the response (*i.e.*, fucoidanase activity), β_0_ is the model intercept, β*_i_* is the linear coefficient, *X_i_* is the level of the independent variable, and *k* is the number of involved variables.

In the present study, six components of the medium were evaluated in twelve experimental trails (Table 2). All the trails were performed in triplicate and the average fucoidanase activity for each trail was used as the response variable. Regression analysis revealed the media components with significant (95% level *p* < 0.05) effect on fucoidanase activity, and these components were evaluated in further optimization experiments.

#### 3.5.2. Response Surface Methodology (RSM)

Based on the selection of the significant variables for fucoidanase production by the PB design experiment, the significant variables were selected, *viz*., wheat bran, kelp powder, and NaCl. Once the ranges of relevant variables were selected, the RSM, using a Box-Behnken design, was used to determine the optimum concentration of these variables for fucoidanase production. In total, 17 experiments were formulated using the statistical software package Design Expert 7.0. The central values of all variables were coded as zero. The minimum and maximum ranges of the variables were used, and the full experimental plan with respect to their values in actual and coded form is provided in Table 3. All the experiments were performed in triplicate and the average of fucoidanase production obtained was considered as the dependent variable or response (Y). The second order polynomial coefficients were calculated and analyzed using the Design Expert 7.0 software statistical package. The general form of the second degree polynomial Equation (4) is:

*Y* = β_0_ + ∑β*_i_X_i_* + ∑β*_ii_X*^2^*_i_* + ∑β*_ij_X_i_X_j_* (4)

where *Y* is the predicted response, *X_i_X_j_* are input variables, which influence the response variable *Y*; β_0_ is the offset term, β*_i_* is the *i*th linear coefficient, β*_ii_* is the *i*th quadratic coefficient, and β*_ij_* is the *ij*th interaction coefficient. However, in this study, the independent variables were coded as *X*_1_, *X*_2_, and *X*_6_*.* Thus, the second order polynomial Equation (5) can be presented as follows:

*Y =* β_0_*+* β_1_*X*_1_*+* β_2_*X*_2_*+* β_6_*X*_6_*+* β_12_*X*_1_*X*_2_*+* β_16_*X*_1_*X*_6_*+* β_26_*X*_2_*X*_6_*+* β_11_*X*^2^_1_*+* β_22_*X*^2^_2_*+* β_66_*X*^2^_6_ (5)

Statistical analysis of the model was performed to evaluate the analysis of variance (ANOVA). This analysis included the Fisher’s *F*-test (overall model significance), its associated probability *p* (*F*), correlation coefficient *R* and determination coefficient *R*^2^, which measure the goodness of fit of the regression model. For each variable, the quadratic models were represented as contour plots (3D) and response surface curves were generated using the Design Expert 7.0 software.

### 3.6. Purification of Fucoidanase

The purification steps were performed at 4 °C and the culture broth was centrifuged at 10,000 rpm for 20 min. The supernatant was filtered through a paper filer and precipitated by adding solid ammonium sulphate. The precipitate was collected by centrifugation at 8000 rpm for 30 min. The precipitate was dissolved and dialyzed against citric acid-sodium citric buffer (0.1 M, pH 6.0). The enzyme solution was loaded on Sephadex G-100 column (2.5 × 100 cm) and eluted at the flow rate of 0.5 mL/min. The fractions were collected and assayed for fucoidanase activity. The fractions exhibiting fucoidanase activity were pooled and used as purified fucoidanase for the biosynthesis of gold nanoparticles.

### 3.7. Biosynthesis and Characterization of Gold Nanoparticles

The purified fucoidanase (1 mL) was added to 10 mL of 1 mM aqueous solution of gold chloride and the solution was kept in a magnetic heater stirrer at 80 °C for 30 min, resulting in a color changing of solution that indicates the formation of gold nanoparticles. The formation of gold nanoparticles was monitored by UV-vis spectroscopy using Beckman Coulter DU530 Life Science UV/vis spectrophotometer (Beckman Coulter, Fullerton, CA, USA). Fourier transform infrared spectroscopy (FTIR) was performed by spectrum GX spectrometry in diffuse reflectance mode operated at a resolution of 4 cm^−1^ of wavelength of about 4000–400 cm^−1^. X-ray diffraction analysis (X’Pert-MPD, Philips, Amsterdam, The Netherlands) was performed by preparing a thin film of powdered gold nanoparticles. The studies on size, morphology and composition of the gold nanoparticles were performed by the means of field emission scanning electron microscopy (FESEM) (JSM-6700, JEOL, Tokyo, Japan) with energy dispersive X-ray analysis (EDXA) and high-resolution transmission electron microscopy (HRTEM) (JEM 1010, JEOL, Japan).

### 3.8. In Vitro Anticancer Activity of Gold Nanoparticles

#### 3.8.1. Cell Viability

Human cervical cancer cell line (HeLa) was purchased from the American Type Culture Collection, Manassas, VA, USA. The cell line was grown in Dulbecco’s Modified Eagle Medium supplemented with 1% penicillin–streptomycin antibiotics and 10% fetal bovine serum. Cancer cells were cultured in 75 cm^2^ cell culture flasks at 37 °C in a 5% CO_2_ atmosphere. HeLa cells were cultured and seeded into 96-well plates approximately as 5 × 10^4^ cells in each well, and were incubated for 48 h [36]. HeLa cells were treated with a series of 50–500 µg/mL concentration of biosynthesized gold nanoparticles. The treated cells were incubated for 24 and 48 h to perform cytotoxic analysis using MTT assay. MTT (3-(4,5-dimethylthiazol-2-yl)-2,5-diphenyltetrazolium bromide, a yellow tetrazole) was prepared at a concentration of 0.5 mg/mL and 100 µL of MTT was added in each well and incubated for 4 h. Purple formazone crystals were observed, and these crystals were dissolved with 100 µL of dimethyl sulfoxide (DMSO), and read at 570 nm in a multi well ELISA plate reader [19]. Optical density value was subjected to the percentage of viability using the following formula.

Percentage of cell viability (%) = OD value of experimental samples/OD value of experimental controls × 100

#### 3.8.2. Morphological Observation

HeLa cells were treated with different concentrations of biosynthesized gold nanoparticles and incubated for 24 and 48 h at 37 °C in a 5% CO_2_ atmosphere. After the incubation of cells, the gross morphological changes in the cell were observed under a Leica DMI3000 B inverted phase contrast microscope (Leica Microsystems GmbH, Wetzlar, Germany).

#### 3.8.3. 4′,6-Diamidino-2-Phenylindole Dihydrochloride (DAPI) Staining

HeLa cells were treated with the above methods for 24 and 48 h, and then fixed with methanol: acetone (3:1, *v*/*v*) prior to washing with phosphate buffered saline. The washed cells were then stained with 1 mg/mL DAPI for 30 min in dark. The nuclear morphology was examined under a fluorescence microscope (Leica Microsystems GmbH, Wetzlar, Germany) to identify cells undergoing apoptosis.

#### 3.8.4. Statistical Analysis

Design Expert 7.0 software was used for data analysis. All experiments were performed in triplicate, and all values were expressed as the mean ± standard deviation (SD). The statistical software, SPSS/14 (one way ANOVA), was used to estimate the statistical parameters.

## 4. Conclusions

Marine actinobacteria, synthesizing rare enzymes, are promising sources of valuable bioactive compounds. Marine actinobacteria are able to degrade the insoluble polysaccharides chitin and agar, as well as the cell-wall polysaccharides of seaweeds and other organisms. The PB design and RSM were employed to optimize medium composition and culture conditions for the production of a novel fucoidanase. The optimal combinations of media constituents and culture conditions for maximum fucoidanase were determined as wheat bran 3.3441 g/L, kelp powder 0.7041 g/L, and NaCl 0.8807 g/L. The fucoidanase yield increased to 16.74 U/mL using the shake-flask system. Optimization of the culture medium and growth conditions reduced the cost of medium components and improved the feasibility of commercial production of fucoidanase. The purified fucoidanase was used for the biosynthesis of gold nanoparticles. To the best of our knowledge, this is the first report on the production and optimization of a novel fucoidanase for the biosynthesis of gold nanoparticles and its cytotoxic effect on HeLa cells. The biosynthesized gold nanoparticles were characterized by UV-vis spectroscopy, XRD, FTIR, FESEM, EDXA, and HRTEM. The cytotoxicity effects of gold nanoparticles on the HeLa cell line were assessed using cell viability and staining techniques, thus, proving that gold nanoparticles exert cytotoxicity effect on HeLa cell lines. Further studies are required to elucidate the precise molecular mechanism involved in cell growth inhibition, thereby permitting the biosynthesized gold nanoparticles as cancer chemopreventive and/or therapeutic agents.
